# Protective effects of pomegranate (*Punica granatum*) juice on testes against carbon tetrachloride intoxication in rats

**DOI:** 10.1186/1472-6882-14-164

**Published:** 2014-05-22

**Authors:** Ebtesam M Al-Olayan, Manal F El-Khadragy, Dina M Metwally, Ahmed E Abdel Moneim

**Affiliations:** 1 Zoology Department, Faculty of Science, King Saud University, Riyadh, KSA; 2 Zoology & Entomology Department, Faculty of Science, Helwan University, Cairo, Egypt; 3 Parasitology Department, Faculty of Veterinary Medicine, Zagazig University, Zagazig, Egypt; 4 Biochemistry and Molecular Biology Department, Asturias Institute of Biotechnology, University of Oviedo, 33006 Oviedo, Spain

**Keywords:** *Punica granatum*, Carbon tetrachloride, Oxidative stress, Testes, Rats

## Abstract

**Background:**

Pomegranate fruit has been extensively used as a natural medicine in many cultures. The present study was aimed at evaluating the protective effects of pomegranate (*Punica granatum*) juice against carbon tetrachloride (CCl_4_)-induced oxidative stress and testes injury in adult Wistar rats.

**Methods:**

Twenty eight Wistar albino male rats were divided equally into 4 groups for the assessment of protective potential of pomegranate juice. Rats of group I (control) received only vehicles and had free access to food and water. Rats of groups II and IV were treated with CCl_4_ (2 ml/kg bwt) via the intraperitoneal route once a week for ten weeks. The pomegranate juice was supplemented via drinking water 2 weeks before and concurrent with CCl_4_ treatment to group IV. Group III was supplemented with pomegranate juice for twelve weeks. The protective effects of pomegranate on serum sex hormones, oxidative markers, activities of antioxidant enzymes and histopathology of testes were determined in CCl_4_-induced reproductive toxicity in rats.

**Results:**

Pomegranate juice showed significant elevation in testosterone, luteinizing hormone (LH) and follicle stimulating hormone (FSH) those depleted by the injection of CCl_4_. Activity levels of endogenous testesticular antioxidant enzymes; superoxide dismutase (SOD), catalase (CAT), glutathione peroxidase (GPx), glutathione-S-transferase (GST) and glutathione reductase (GR) and glutathione (GSH) contents were increased while lipid peroxidation (LPO) and nitric oxide (NO) were decreased with pomegranate juice. Moreover, degeneration of germ and Leydig cells along with deformities in spermatogenesis induced after CCl_4_ injections were restored with the treatment of pomegranate juice.

**Conclusion:**

The results clearly demonstrated that pomegranate juice augments the antioxidant defense mechanism against carbon tetrachloride-induced reproductive toxicity and provides evidence that it may have a therapeutic role in free radical mediated diseases.

## Background

Male sexual dysfunction composed of several problems associated with sperm concentration, motility and hormonal imbalance e.g., low testosterone level, which are caused by alcoholism, drug abuse, aging and cigarette smoking, anti depressant drugs and exposure of toxic chemicals [1,2]. Furthermore, an increase in oxidative damage to sperm membranes, proteins and DNA is associated with alterations in signal transduction mechanisms that affect fertility [3].

Carbon tetrachloride (CCl_4_), an industrial solvent, is an extensively used xenobiotic to induce chemical liver injury. CCl_4_ is metabolized by hepatic microsomal cytochrome P450 to trichloromethyl free radical. Trichloromethyl can react with sulfhydryl groups (glutathione and protein thiols) and antioxidant enzymes such as catalase and superoxide dismutase. Over production of trichloromethyl free radicals initiate a membrane lipid peroxidation, eventually leading to various pathological changes [4]. Studies using antioxidants indicate the role of oxidative stress in CCl_4_-induced testes injury [5]. Furthermore, some studies have revealed that natural products, containing antioxidant, protect the testes against lipid peroxidation and impairment in antioxidant status induced by CCl_4 _[3,4].

Pomegranate (*Punica granatum*) has been acclaimed for its health benefits, this fruit has long been cultivated and consumed as a fresh fruit or in beverage form especially in the Mediterranean region. Pomegranate fruit, juice and peel possess a marked antioxidant capacity [6] with a high content in polyphenols, in particular, ellagitannins, condensed tannins and anthocyanins [7]. Some of these antioxidant molecules have been shown to be bioavailable and safe [8]. Pomegranate juice consumption increases significantly sperm quality, spermatogenic cell density, antioxidant activity and testosterone level in male rats [9]. In addition, pomegranate juice has been proposed as chemopreventive, chemotherapeutic, antiatherosclerotic and antiinflammatory [10-12] and accordingly its consumption has grown tremendously [8,13]. So, it would be important to confirm the antioxidant effect of pomegranate. Therefore, this study was performed in order to investigate the protective effects of pomegranate juice on CCl_4_-induced oxidative stress and testes injury in adult Wistar rats.

## Methods

### Chemicals

Carbon tetrachloride (CCl4; CAS Number 56-23-5) and Tris–HCl buffer were purchased from Sigma (St. Louis, MO, USA). Perchloric acid, thiobarbituric acid (TBA) and trichloroacetic acid (TCA) were purchased from Merck. All other chemicals and reagents used in this study were of analytical grade. Double-distilled water was used as the solvent.

### Animals

Twenty four adult male Wistar albino rats weighing 200-250 g (9-10 weeks) were obtained from The Holding Company for Biological Products and Vaccines (VACSERA, Cairo, Egypt). The animals were kept in wire bottomed cages in a room under standard condition of illumination with a 12-hours light-dark cycle at 25 ± 1°C for one week until the beginning of treatment. They were provided with tap water and balanced diet ad *libitum*.

All experimental procedures involving animals were conducted in accordance with the guidelines of the National Program for Science and Technology of Faculty of Science, King Saud University. The study protocol was approved (No. 1/3/12337) by Ethical Committee of King Saud University (KSU), Riyadh, of the joined work between College of Science (KSU) and Zoology Department (Helwan University).

### Plant material

*Punica granatum* fruits were collected from market of East Cairo, Egypt in the months of February-March, 2012. The plant material was authenticated in Botany Department, Faculty of Science, Helwan University, Cairo-Egypt on the basis of taxonomic characters and by direct comparison with the herbarium specimens available at the herbarium of the Botany Department.

### Pomegranate juice preparation

Ten kg of pomegranates were washed and manually peeled, without separating the seeds. Juice was obtained using a commercial blender (Braun, Germany), filtrated with a buchner funnel and immediately diluted with distilled water to volume of 1:3 and stored at -20°C for no longer than 2 months [13].

### Pomegranate juice stability

Pomegranate juice stability was assessed by measuring initial total phenolic content and evaluating the alterations after 2 and 3 days of exposure to the same conditions as the juice supplied to the animals. The total polyphenol content of the pomegranate juice was 74.8 μg gallic acid equivalent/ml juice, determined following the standard Folin-Ciocalteu method. The content of polyphenol was not markedly affected after long time storage.

### HPLC-ESI-MS analysis

Three replicates from juice were centrifuged in an eppendorf tube (2 min at 1400 rpm) and filtered through a 0.45 μm filter. A liquid chromatography apparatus 1290 series from Agilent Technologies, including a degasser, a binary pump delivery system, and an automatic liquid sampler, was used and coupled to Agilent Triple Quad Model 6460 mass spectrometer detectors. The HPLC column was a ZORBAX Eclipse plus C18 (4.6 × 150 mm, 5 μm) from Agilent Technologies (Agilent Technologies, Palo Alto, CA, USA). Separation was carried out by acetic acid (2%; A) and acetonitrile (B). The following multistep linear gradient was applied: 0 min, 5% B; 2 min, 7% B; 4 min, 9% B; 6 min, 12% B; 8 min, 15% B; 9 min, 16% B; 10 min, 17% B; 11 min, 17.5% B; 12 min, 18% B; 14 min, 20% B; 16 min, 28% B; 18 min, 100% B; 22 min, 100% B; 23 min, 5% B. The initial conditions were maintained for 5 min. The flow rate was set at 0.80 ml/min throughout the gradient. The injection volume in the HPLC system was 2.5 μl. The chromatograms were registered at 280 and 360 nm. Separation was carried out at 30°C. MS analysis was carried out using electrospray ionization (ESI) interface in negative ionization mode.

### Experimental protocol

To study the protective effects of pomegranate on carbon tetrachloride mediated reproductive toxicity, twenty eight adult male rats were randomly allocated to four groups of seven rats of each. Group I (Con) served as control and received 300 μl of saline by intraperitoneal (i.p.) injection route each week. Group II (CCl_4_) received weekly i.p. injection of 2 ml CCl_4_/kg body weight (bwt) for 10 weeks as described by sohn et al. [14]. Group III (Pom) received juice supplied on dark water bottles and renewed every 2–3 days [13] and the animals of group IV (Pom + CCl_4_) received pomegranate juice as group III for 2 weeks before and concurrent with CCl_4_ treatment that injected intraperitoneally for 10 weeks at a dose of 2 ml of CCl_4_ per kg bwt. After one week of the last i.p. injection of CCl_4_, blood samples were collected from all animals by cardiac puncture (under anaesthesia with chloroform). Right testes was promptly excised, weighed and homogenized immediately to give 50% (w/v) homogenate in ice-cold medium containing 50 mM Tris–HCl, pH, 7.4. The homogenate was centrifuged at 3000 rpm for 10 min at 4°C. The supernatant (10%) was used for the various biochemical determinations.

### Testes index

Relative weight of testes was calculated as left testes weight/body weight × 100.

### Biochemical estimations

#### Oxidative stress

Homogenates of testes were used to determine lipid peroxidation (LPO) by reaction of thiobarbituric acid (TBA) [15]. Similarly, those homogenates were used to determine nitrite/nitrate (nitric oxide; NO) [16] and glutathione [17].

### Enzymatic antioxidant status

Homogenates of testes were used in determination of superoxide dismutase (SOD) [18], catalase (CAT) [19], glutathione peroxidase (GPx) [20], glutathione-S-transferase (GST) [21] and glutathione reductase (GR) [22].

### Estimation of serum testosterone, luteinizing hormone and follicle stimulating hormone

Quantitative measurement of serum testosterone, follicle stimulating hormone (FSH) and luteinizing hormone (LH) were carried out adopting ELISA technique using kits specific for rats purchased from BioVendor (Gunma, Japan) according to the protocol provided with each kit.

### Histological examination

The testes tissues were collected and immediately fixed with 10% buffered formalin, and embedded in paraffin. Sections (4–5 μm) were prepared and then stained with hematoxylin and eosin dye for photomicroscopic observations.

### Statistical analysis

Results were expressed as the mean ± standard error of the mean (SEM). Data for multiple variable comparisons were analyzed by one-way analysis of variance (ANOVA). For the comparison of significance between groups, Duncan’s test was used as a *post hoc* test according to the statistical package program (SPSS version 17.0) and figures were drawn with Origin (version 8). All *p* values are two-tailed and *p* < 0.05 was considered as significant for all statistical analysis in this study.

## Results

### HPLC-ESI-MS results

**Figure 1:**
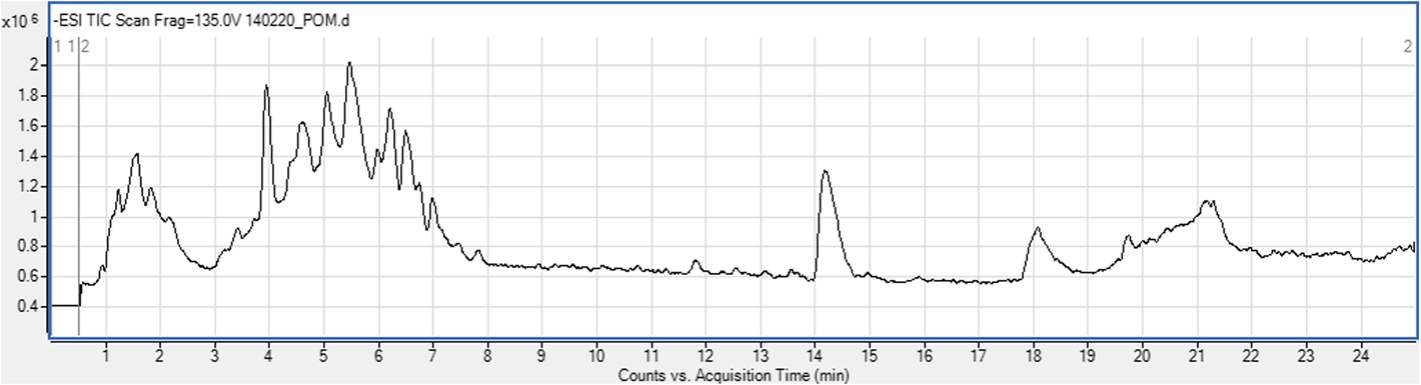
**HPLC-ESI-MS chromatogram of *P. granatum *** **juice.**

The phytochemical fingerprint of pomegranate juice was determined using a electrospray ionization mass spectrometry (ESI-MS). HPLC-MS technique is an important method used for identifying complex mixtures, especially the phenolics or its fraction found in the plant, either by using standard compounds (target identification) or by comparing mass spectrum obtained with literatures (tentative identification). This method is useful to avoid replication, safe time, and money used in isolation and identification of known compounds. In the current study, pomegranate juice was subjected to HPLC-ESI-MS analysis. HPLC-ESI-MS experiment allowed the identification of a total of 41 compounds. Hydrolyzable tannins were the main class of (poly)phenolics identified in pomegranate juice. A broad number of anthocyanins, non-coloured flavonoids and phenolic acids were also found. Other phytochemicals, such as lignans, were also observed. Likewise, several organic acids were detected (Figure 1).

The 41 compounds were identified by the interpretation of their fragmentation patterns obtained from the mass spectrum. Data provided by literature information was employed for the comprehensive evaluation of the juice. The retention times and mass spectrum data along with peak assignments for compounds identified using negative ionization are described in Additional file 1: Table S1. As shown in Additional file 1: Table S1, m/z 301 (-) ions from MS analysis are evidence for the presence of ellagic acid precursor and HHDP in the molecules. Ellagitannins were also detected in the pomegranate juice assessed. They were distinguished by their characteristic fragment ion spectra yielding sequential losses of galloyl (m/z 152), gallate (m/z 170). Gallotannins, composed by monomeric and dimeric galloyl moieties linked to a hexose sugar were also detected. Six compounds matching the molecular ion m/z 331 were observed and considered as gallotannins. The different flavonoids belonging to four subclasses of non-coloured flavonoids (flavan-3-ols, flavonols, dihydrochalcones and flavanones) were detected. The flavan-3-ols detected was (+)-gallocatechin. Kaempferol, phlorizin and quercetin displayed flavonols, dihydrochalcones and flavanones subclasses, respectively. A total of three phenolic acid derivatives were found in pomegranate juice in this study, vanillic acid, its hexoside and syringetin-hexoside. In chromatographic analysis, organic acids were separated and identified by comparison with published data. Citric acid and its derivative have been pointed out as the main organic acids in pomegranate juices. As summarized in Additional file 1: Table S1, six anthocyanins were detected. Anthocyanins are the phenolics responsible for the red colour of pomegranate juice. The anthocyanin profile comprised cyanidin, pelargonidin, and delphinidin.

Additionally, gas chromatography–mass spectrometry (GC-MS) analysis experiment was performed. GC-MS chromatogram of the pomegranate juice showed 33 peaks indicating the presence of 33 phytochemical constituents (Additional file 2: Figure S1 and Table S2).

### Testes index

**Figure 2:**
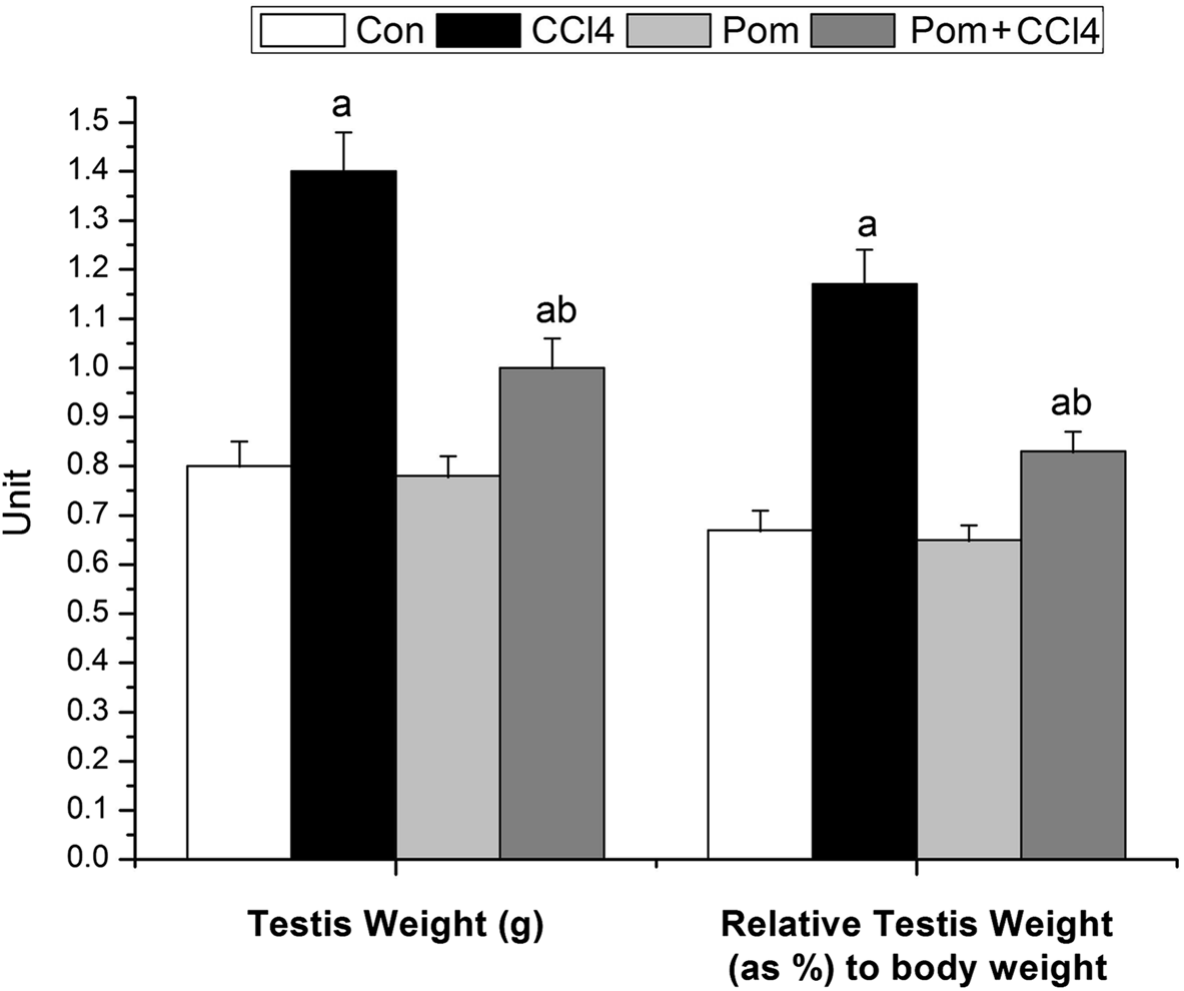
**Effect of *P. granatum *juice on testes weight and relative testes body weight of rats treated with CCl**_**4**_**.** Values are means ± SEM (n = 7). a: significant change at *p* < 0.05 with respect to the **Con** group. b: significant change at *p* < 0.05 with respect to the **CCl**_**4**_ group.

The toxicity of CCl_4_ on testes weight and the relative testes weight were represented in Figure 2. The injection of rats with CCl_4_ caused a significant increase in testes weight (1.4 g; *p* < 0.05) and relative testes weight by 74.63% comparing to the control group. Treatment with pomegranate juice erased the CCl_4_ toxicity and significantly improved testes weight and relative testes weight when compared with the CCl_4_ group which helps in reducing edema in the testes due to fluid accumulation, however, pomegranate juice failed to return the testes weight and relative testes weight (1 g and 23.88%, respectively) to the control values. Supplementation of pomegranate juice on its own did not change the testes weight (0.78 g) and relative testes weight as compared with that in the control group.

### Redox status

**Figure 3:**
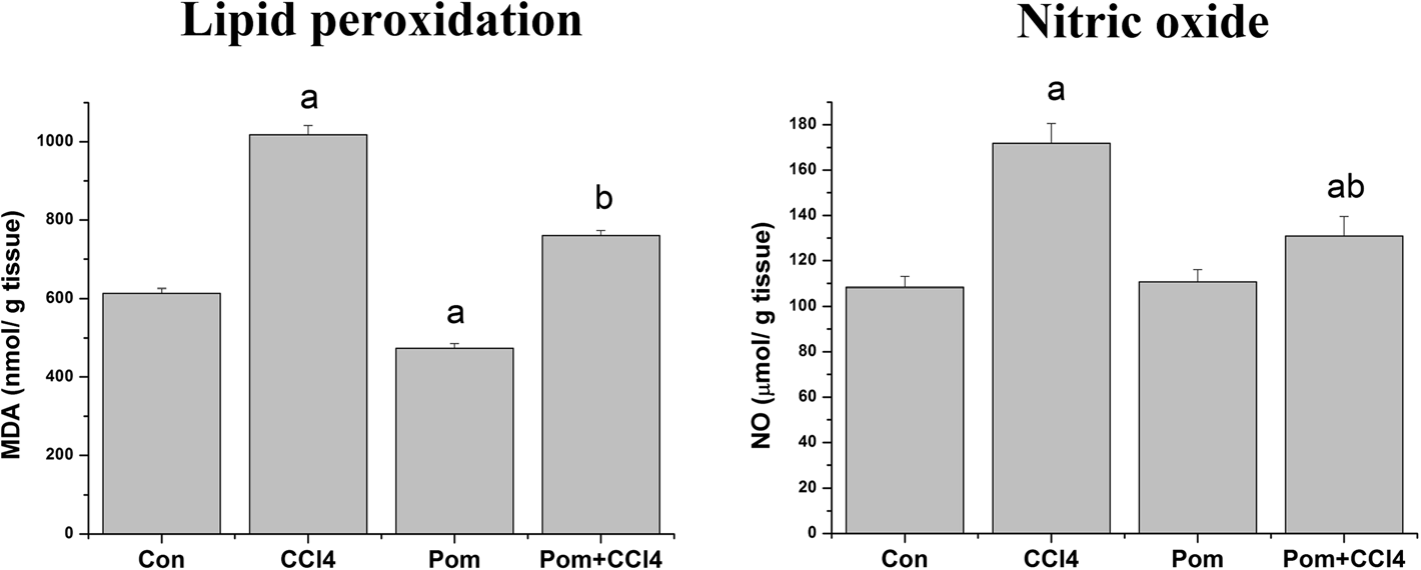
**Effect of *P. granatum *juice on lipid peroxidation, expressed as malondialdehyde (MDA), and nitric oxide (NO) contents in testes of rats treated with CCl**_**4**_**.** Values are means ± SEM (n = 7). a: significant change at *p* < 0.05 with respect to the **Con** group. b: significant change at *p* < 0.05 with respect to the **CCl**_**4**_ group.

The LPO level is widely used as a marker of free-radical mediated lipid peroxidation. The results of the LPO assays in the testes homogenates are shown in Figure 3. LPO level in the CCl_4_-treated group (65.9%) was significantly higher than in the vehicle-control group. Supplementation of pomegranate juice significantly decreased CCl_4_-induced testisticular lipid peroxidation. NO reacts with O_2_^•-^ and leads to the formation of ONOO^-^ (peroxynitrite), which contributes to reproductive toxicity due to its cytotoxicity properties. CCl_4_ injection caused a significant increase (58.4%; *p* < 0.05) in NO content in testes homogenates compared to the control group. This oxidant molecule was significantly reduced (20.8%) when animals were supplemented with pomegranate juice showing the ameliorative effects of pomegranate juice (Figure 3).

Antioxidant enzymes such as CAT, SOD, GPx, GR and GST, as well as, glutathione as a non-enzymatic antioxidant substance were estimated in the present study. There was a significant decrease in GSH in the testes homogenates of CCl_4_ group as compared to the control group (-22.90% at *p* < 0.05). The supplementation of rats with pomegranate juice pre- and concurrent with CCl_4_ injection caused a significant increase in GSH not only when compared with CCl_4_ group but also with the control group (Table 1). GPx, GR and GST activities were also significantly decreased in the testisticular tissues of rats inoculated with CCl_4_ (Table 1), but pomegranate juice was able to significantly elevate these parameters after 10 weeks of CCl_4_ injection.

**Table 1:** **Protective roles of *P. granatum *juice on glutathione (GSH) content and glutathione-S-transferase (GST), glutathione peroxidase (GPx) and glutathione reductase (GR) activities on testes of rats treated with CCl**_**4**_

**Groups**	**GSH (mmol/g tissue)**	**GST (μmol/h/g tissue)**	**GPx (U/g tissue)**	**GR (μmol/g tissue)**
**Con**	18.08 ± 0.65	0.31 ± 0.02	1144.32 ± 66.07	7.23 ± 0.39
**CCl**_**4**_	13.94 ± 1.11^a^	0.12 ± 0.01^a^	736.33 ± 39.75^a^	5.22 ± 0.38^a^
**Pom**	32.72 ± 0.93^a^	0.42 ± 0.01^a^	1155.04 ± 92.86	7.51 ± 0.34
**Pom + CCl**_**4**_	22.41 ± 0.71^ab^	0.16 ± 0.01^a^	856.87 ± 63.50^a^	5.69 ± 0.19^a^

Values are means ± SEM (n = 7). ^a^: significant change at *p* < 0.05 with respect to the **Con** group, ^b^: significant change at *p* < 0.05 with respect to **CCl**_**4**_ group.

**Figure 4:**
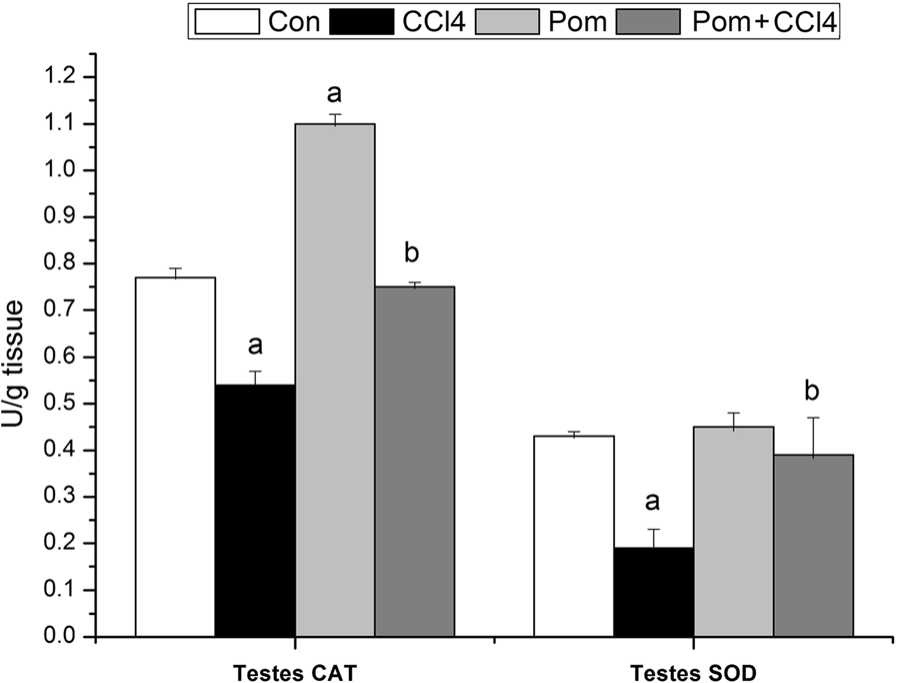
**Effect of *P. granatum *juice on superoxide dismutase (SOD) and catalase (CAT) activities in testes of rats treated with CCl**_**4**_**.** Values are means ± SEM (n = 7). a: significant change at *p* < 0.05 with respect to the **Con** group. b: significant change at *p* < 0.05 with respect to the **CCl**_**4**_ group.

Carbon tetrachloride decreased the activities of SOD and CAT to approximately -55.81% and -29.87%, respectively, compared to the control group (Figure 4). Rats supplemented with pomegranate together with CCl_4_ for 12 weeks experienced a significant increase in SOD and CAT compared to the CCl_4_ group (Figure 4).

### Hormonal fertility studies

**Figure 5:**
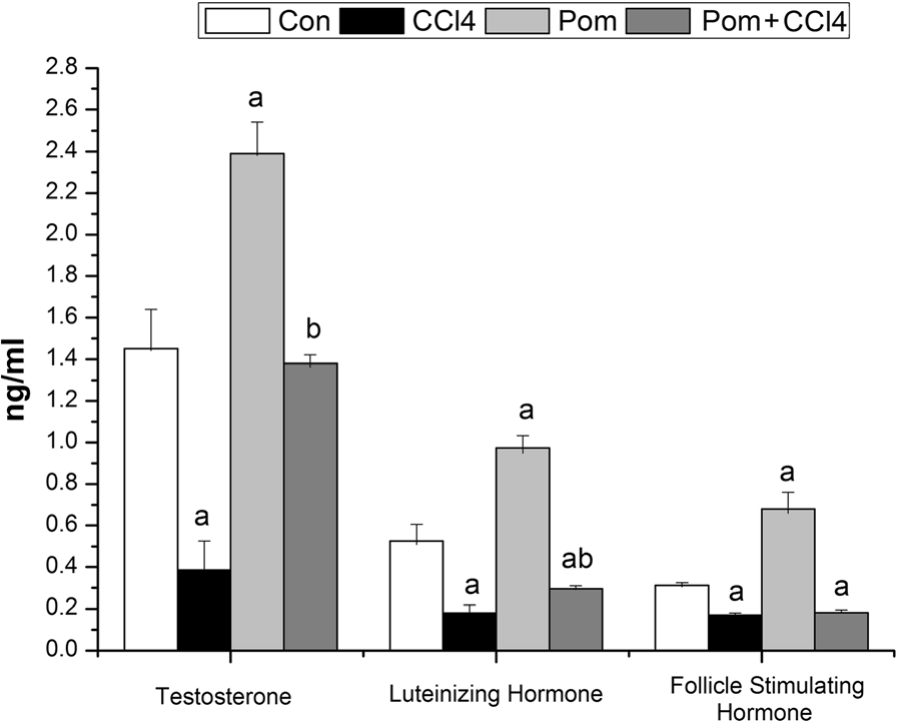
**Effect of *P. granatum *juice on serum testosterone, luteinizing hormone and follicle stimulating hormone in rats treated with CCl**_**4**_**.** Values are means ± SEM (n = 7). a: significant change at *p* < 0.05 with respect to the **Con** group. b: significant change at *p* < 0.05 with respect to the **CCl**_**4**_ group.

The mean values of the serum hormones; testosterone, luteinizing hormone and follicle stimulating hormone are shown in Figure 5. After treatment of rats with CCl_4_ for 10 weeks, the mean values of testosterone, LH and FSH were decreased as compared to the control group (-72.1%, -65.4% and -19.0%, respectively). In the (Pom + CCl_4_) treated group, testosterone levels were restored to control values. Serum level of LH in this group was increased significantly as compared with the CCl_4_-treated group (*p* < 0.05), but it still decreased significantly compared to the control group. Mean values of FSH in the (Pom + CCl_4_) treated group did not differ from the CCl_4_-treated group. Statistically significant increase in the levels of testosterone, LH and FSH were observed in rats treated with pomegranate juice on its own as compared to the control group.

### Histological studies

**Figure 6:**
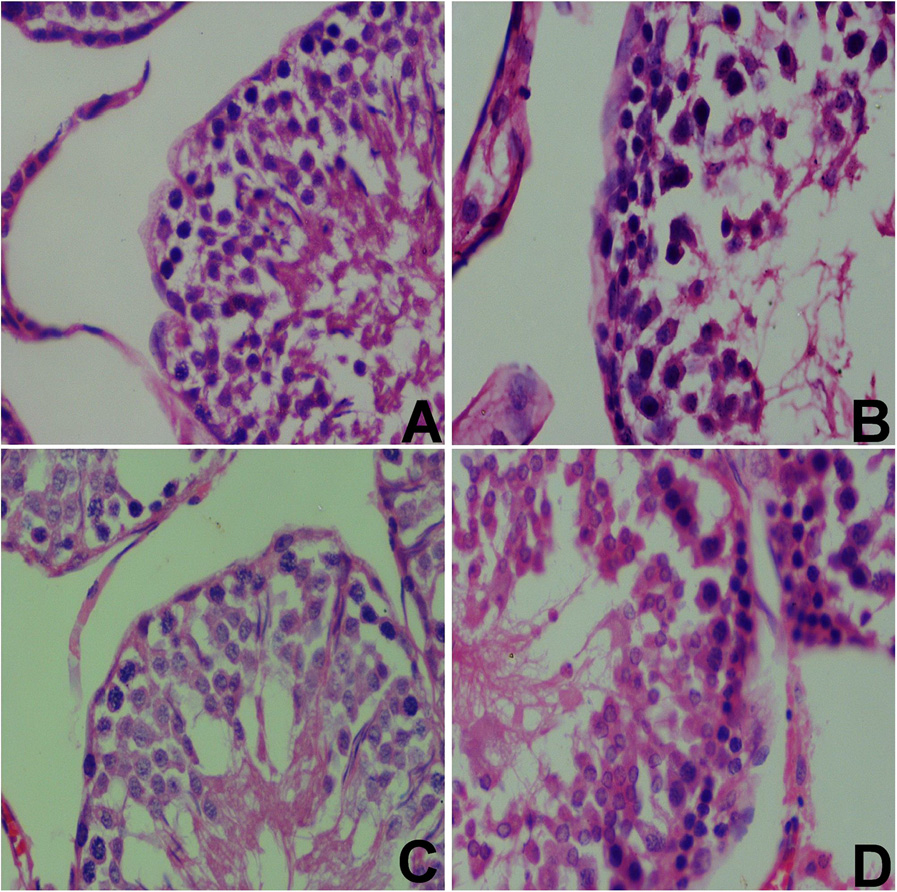
**Histological changes in the testes of rats. (A)** Control testes with normal spermatogenesis. **(B)** Rats treated with CCl_4_ with prominent inflammation, complete swallowing of seminiferous tubules and degenerated germ cells. **(C)** Rats supplemented with Pom. **(D)** Rats treated with the Pom and CCl_4_. Sections were stained with hematoxylin and eosin (400x).

Among rats given daily pomegranate juice supplements alone, there were no marked changes in testicular histology relative to controls (Figure 6C). Thus normal spermatogenesis, well preserved Sertoli cells and well delineated tubular basement membrane were observed. The interstitium between tubules and Leydig cells was also intact. However, in the CCl_4_-treated group, differences were observed in the histology of testes, where complete swallowing of seminiferous tubules was exhibited while in other areas of the section the tubular basement membranes of seminiferous tubules were identified, but most of the germ cells were degenerated, especially the ones involving highly differentiated germ cells along with deformed sperm. The ground substance within the interstitium also partially disappeared and was replaced by fibroblast and inflammatory cells (Figure 6B). In the (Pom + CCl_4_)-treated group, those toxic effects were ameliorated (Figure 6D).

## Discussion

Our study was focused on the protective effect of pomegranate juice against CCl_4_-induced reproductive toxicity in male rats. Pomegranate has a protective effect due to its active ingredients like tannins [23,24], phenolic acids [25], estrogenic flavonoids [26] and conjugated fatty acids [27], these compounds are found in substantial amounts in the peels, juice and seed oil of the pomegranate fruits [28].

Our results with the pomegranate juice agree with previous reports showing that some flavonoids from plants and fruits were potent O_2_^•-^ scavengers [29]. Moreover, several studies have shown that the pharmacological effects of flavonoids are related to their antioxidant activity, which can be due to their ability to scavenge OH^•^ and O_2_^•-^, to chelate metal ions and to exert a synergistic effects with other antioxidant metabolites [30-32]. Our results in conjunction with the others mentioned above, suggest that flavonoids could constitute one of the active components of *P. granatum*.

The basis of CCl_4_ testisticular toxicity lies in its biotransformation by the cytochrome P-450 system giving the trichloromethyl radical (CCl_3_^•^) which is further converting to the trichloromethyl peroxyl radical (CCl_3_O_2_^•^). CCl_4_ metabolites react with polyunsaturated fatty acids and form covalent adducts with lipids and proteins. These events lead to lipid peroxide formation and destruction of cell membranes with the consequent testis injury [33].

The NO radicals play an important role in inducing an inflammatory response and their toxicity multiplies only when they react with O_2_^•-^ radicals to form peroxynitrite, that damages biomolecules such as proteins, lipids and nucleic acids [34,35]. In our results, pomegranate juice was active and it may possess very potent and novel therapeutic agents for the scavenging of NO. This juice may also exert their effects on the regulation of pathological conditions caused by excessive generation of NO and its oxidation product-peroxynitrite.

It has been reported that SOD, CAT and GST constitute a mutually supportive defense system against ROS [36-38]. In the present study, we demonstrated that CCl_4_ induced a significant decrease in the activities of antioxidant enzymes namely, CAT, SOD, GPx, GR and GST. The inhibitions in antioxidant enzymes after CCl_4_ injection are probably due to protein inactivation by ROS. Oxidative damage often leads to the loss of specific protein function [39]. The inter-relationships between protein oxidation, protein dysfunction and diseases are still unclear, but it is known that oxidative changes in enzymes and structural proteins play a significant role in the pathophysiology of many diseases such as Parkinson’s disease [40] and seizures [41].

In fact, a decrease of testisticular GST activity in CCl_4_-treated rodents has been reported before [42]. A decrease in GST activity during CCl_4_ toxicity might be due to the decreased availability of GSH during enhanced lipid peroxidation. The inhibition of SOD in testes in CCl_4_-treated rats may be due to the enhanced lipid peroxidation or inactivation of antioxidant enzymes. This would cause an increased accumulation of superoxide radicals, which could further stimulate lipid peroxidation. The pomegranate juice was able to partially prevent CCl_4_-induced decay of antioxidant enzyme activities; this preventive effect was also observed at the histological level (Figure 6). A similar scavenger role of flavonoids in mice and rats after exposure to CCl_4_ had been determined in liver [43].

Alterations in the spermatogenic cycle and degeneration in seminiferous tubules has been reported with CCl_4_ in rats [44]. In the CCl_4_-treated group, seminiferous tubules and germ cells were degenerated, interstitium partially disappeared and was replaced by fibroblast and inflammatory cells in some of the areas of testes. The presence of polyphenols and flavonoids in *P. granatum* might be involved in ameliorating the effects of CCl_4_-induced toxicity [45]. These constituents of pomegranate juice are thought to provide many beneficial effects against organ damages.

In the present study, CCl_4_ treatment decreased the serum level of testosterone, FSH and LH. Secretion of testosterone is probably impaired due to excessive oxidative stress and the degeneration of Leydig cells [46]. Metabolites of testosterone reciprocally depress FSH and LH secretion [47]. Injuries in germinal epithelial caused with CCl_4_ treatment can partially stimulate spermatogenesis that may occur due to less production of androgen binding proteins. The toxic effects of CCl_4_ might affect the suprachiasmatic hypothalamic nucleus (SCN) that may result in the failure of pituitary to secrete FSH and LH and will result in testicular dysfunction leading to infertility [48]. Treatment of rats with pomegranate juice ameliorated the toxic effects of CCl_4_ and the levels of testosterone, FSH and LH were increased. Pomegranate contains tannins, phenols and flavonoids which can directly or indirectly reduce oxidative damage by preventing the excessive generation of free radicals. In addition, treatment of rats with pomegranate juice alone, slightly increases the serum levels of testosterone, FSH and LH. The increase in sex hormones in the present study due to the pomegranate juice can be in part due to the ability of pomegranate to reduce stress hormones, such as cortisol, as seen by Hong et al. [49].

## Conclusion

The results clearly demonstrate that pomegranate juice augments the antioxidants defense mechanism against carbon tetrachloride-induced reproductive toxicity and provides evidence that it may have a therapeutic role in free radical mediated diseases. Our results show that the protective effect of pomegranate may be due to both an increase in the activity of the antioxidant-defense system and an inhibition of lipid peroxidation and nitric oxide production.

## Abbreviations

CCl4: Carbon tetrachloride; ROS: Reactive oxygen species; LPO: Lipid peroxidation; NO: Nitric oxide; GSH: Glutathione; SOD: Superoxide dismutase; CAT: Catalase; GPx: Glutathione peroxidase; GST: Glutathione-S-transferase; GR: Glutathione reductase; FSH: Follicle stimulating hormone; LH: Luteinizing hormone.

## Competing interests

The authors declared that they have no competing interests.

## Authors’ contributions

AA made a significant contribution to conception and design of the study, acquisition and analyses of data and drafting of the manuscript. ME, DM and EA made contribution in sample collection and design. All the authors read the revised manuscript and approved.

## Pre-publication history

The pre-publication history for this paper can be accessed here:

http://www.biomedcentral.com/1472-6882/14/164/prepub

## Supplementary Material

Additional file 1: Table S1 Identification of phytochemical compounds by HPLC-ESI-MS in pomegranate juice. Click here for file

Additional file 2: Figure S1 GC-MS analysis of pomegranate juice. **Table S2.** Identification of phytochemical compounds by GC-MS in pomegranate juice. Click here for file
