# Supplementary material for: Protective effects of pomegranate (Punica granatum) juice on testes against carbon tetrachloride intoxication in rats
Source: BMC Complement Altern Med. 2014 May 22;14:164. doi: 10.1186/1472-6882-14-164 (PMC4041339; doi:10.1186/1472-6882-14-164)
Supplement: Additional file 1: Table S1 — Identification of phytochemical compounds by HPLC-ESI-MS in pomegranate juice. [file 1472-6882-14-164-S1.doc]

**Additional file 1: Table S1** Identification of phytochemical compounds by HPLC-ESI-MS in pomegranate juice.

|  | **Assignment** | **RT** | **[M – H]^−^**  **(m/z)** | **MS**  **(m/z)** |
| --- | --- | --- | --- | --- |
|  | Citric acid derivative | 0.93 | 399 | 111, 177, 191 |
|  | Cyanidin-3-glycoside | 1.02 | 445.3 | 181, 283, 367 |
|  | Citric acid | 1.31 | 191 | 111, 171 |
|  | Galloyl-glucose | 1.58 | 331 | 152, 169 |
|  | Cyanidin-pentoside-hexoside | 2.19 | 582 | 181, 265 |
|  | Delphinidin-3-glycoside | 3.18 | 463 | 305 |
|  | Hexahydroxydiphenoyl(HHDP)-hexoside | 3.37 | 483 | 177, 301 |
|  | Caffeic acid glucoside | 3.62 | 343 | 177 |
|  | Di(HHDP-galloylglucose)-pentose | 3.73 | 707 | 169, 301, 495, 593 |
|  | Caffeic acid hex derivative | 3.93 | 541 | 177, 305, 343 |
|  | Punicalagin α | 4.24 | 1053 | 301, 601, 779 |
|  | Punigluconin | 4.43 | 799 | 289, 453, 541, 633, 707 |
|  | Ellagic acid derivative | 4.84 | 391 | 125, 175 |
|  | Ellagic acid deoxyhexose | 5.06 | 447 | 183, 301 |
|  | Ellagic acid glucoside | 5.18 | 463 | 301 |
|  | Digalloyl-HHDP-hexoside (pedunculagin II) | 5.46 | 783 | 170, 301, 447 |
|  | Flavogalloyl-HHDP-gluconic acid (lagerstannin B) | 5.55 | 949 | 170, 301, 565, 641 |
|  | Gallocatechin-pelargonidin-3-hexoside | 5.99 | 744 | 152, 331, 675 |
|  | Gallic acid | 6.22 | 170 | 152 |
|  | Pelargonidin-3,5-diglycoside | 6.50 | 593 | 211, 301, 495 |
|  | Pedunculagin I isomer | 6.67 | 783 | 152, 257, 301 |
|  | Galloyl-bis-HHDP-hexoside (casuarinin) | 6.99 | 937 | 170, 301, 541, 659 |
|  | Gallagyl-glucoside (punicalin) | 7.36 | 783 | 170, 331, 481, 649 |
|  | Dihydrokaempferol-hexoside | 7.85 | 449 | 255, 284, 327, 447 |
|  | Ellagic acid-hexoside | 8.47 | 463 | 229, 257, 301 |
|  | Vanillic acid | 10.36 | 167 | 125, 152 |
|  | Quercetin-hexoside | 10.74 | 463 | 301 |
|  | Punicalagin β | 11.81 | 1056 | 170, 301, 601, 779 |
|  | Kaempferol-hexoside | 12.55 | 447 | 255, 284, 327 |
|  | Punicalagin isomers | 13.10 | 541 | 170, 229, 257, 301 |
|  | Kaempferol rutinoside | 14.17 | 593 | 255, 284, 331, 447 |
|  | Galloyl-HHDP-glucoside (lagerstannin C) | 15.85 | 648 | 170, 301, 331 |
|  | Galloyl-HHDP-hexose | 18.05 | 633 | 152, 331, 453, 541 |
|  | Granatin B | 19.71 | 951 | 301, 513, 615, 783 |
|  | Vanillic acid-hexoside | 20.43 | 328 | 125, 152, 167 |
|  | Peduncalagin I | 20.68 | 783 | 301, 463 |
|  | Lagerstannin B derivative | 21.01 | 961 | 175, 355, 489, 773 |
|  | Cyanidin-3-rutinoside | 21.33 | 593 | 288, 450 |
|  | Syringetin-hexoside | 22.73 | 505 | 181, 283, 367, 445 |
|  | Phlorizin | 23.52 | 439 | 191, 290, 379 |
|  | Castalagin derivative | 24.59 | 958 | 301, 675, 779 |

Abbreviations: HHDP, hexahydroxydiphenoyl; RT, retention time.
