# Supplementary material for: Protective effects of pomegranate (Punica granatum) juice on testes against carbon tetrachloride intoxication in rats
Source: BMC Complement Altern Med. 2014 May 22;14:164. doi: 10.1186/1472-6882-14-164 (PMC4041339; doi:10.1186/1472-6882-14-164)
Supplement: Additional file 2: Figure S1 — GC-MS analysis of pomegranate juice. Table S2. Identification of phytochemical compounds by GC-MS in pomegranate juice. [file 1472-6882-14-164-S2.doc]

**GC-MS analysis of pomegranate juice**

**Materials and methods**

**Pomegranate juice preparation**

##### Ten kg of pomegranates were washed and manually peeled, without separating the seeds. Juice was obtained using a commercial blender (Braun, Germany), filtrated with a buchner funnel and immediately diluted with distilled water to volume of 1:3 and stored at -20 ºC for no longer than 2 months [1].

##### Gas Chromatography – Mass Spectrometry (GC–MS) Analysis

The GC-MS analysis of pomegranate juice was performed with Thermo Scientific, Trace GC Ultra & ISQ Single Quadruple MS. The inert gas helium (99.9995%) was used as carrier gas, at flow rate of 1.5 ml /min, split ratio 10:1; sample size, 1μl injected using the splitless injection technique; fused capillary silica column TG-5MS (30m × 0.25mm × 0.25µm). Temperatures: injector: 260 ºC, detector: 300 ºC, column: 70 ºC, 10 ºC min^−1^, 260 ºC (10min). The total GC running time is at 60 min. The MS was taken at 70 eV. The MS scan parameters included a mass range of m/z 40-1000, a scan interval of 0.5 s, a scan speed of 2000 amu s^−1^, and a detector voltage of 1.0 kV. Identification of compounds was conducted using the database of Wiley9 combined with NIST 11 mass spectral database. The name, molecular weight, molecular formula and area under peak of the components of the test materials were ascertained.

**Results**

GC-MS chromatogram of the pomegranate juice (Figure S1) showed 33 peaks indicating the presence of 33 phytochemical constituents. On comparison of the mass spectra of the constituents with Wiley9 combined with NIST 11 mass spectral database, the 33 phytoconstituents were characterized and identified (Table S1). The major phytochemical constituent’s mass spectra are 3,5-Dihydroxy-6-methyl-2,3-dihydro-4H-pyran-4-one (5.76%), 1,3-Cyclopentadiene,5-(1-methylethylidene) (0.47%), D-Fructose, diethyl mercaptal, pentaacetate (0.35%), Pyrogallol (3.48%), 1,3-Dioxocane,2-pentadecyl (0.55%), α-Pinene (0.68%), 18,19-Secoyohimban-19-oic acid,16,17,20,21-tetradehydro16-(hydroxymethyl), methyl ester, (15á,16E) (0.38%), γ-Terpinene (5.43%), Methyl-9,9,10,10-D4-octadecanoate (1.82%), Isochiapin A (0.82%), Androsta-1,4-dien-3-one (0.32%), 2-[5-(2-Methoxy-5-nitro-phenyl)-furan-2-ylmethylene]-6,8-dimethyl-benzo[4,5]imidazo[2,1-b]thiazol-3-one (0.77%), 4,8-Dimethyl-5'-(morpholinomethyl)-4',5'-dihydro psoralen (0.86%), Octanoic acid (1.37%), Docosane (1.13%), Cyclopentadecanol (1.70%), Z-10-Methyl-11-tetradecen-1-ol propionate (0.39%), Hexadecanoic acid, methyl ester (5.36%), Hexadecanoic acid-2,3-dihydroxypropyl ester (1.52%), 4-Piperidine acetic acid,1-acetyl-5-ethyl-2-[3(2-hydroxyethyl)-1H-indol-2-yl]-à-methyl, methyl ester (0.84%), 9,12-Octadecadienoic acid, methyl ester (0.83%), 9,12,15-Octadecatrienoic acid, methyl ester (15.64%), Phytol (22.54%), Octadecanoic acid, methyl ester (0.60%), Flavones 4’-OH,5-OH,7-di-O-glucoside (Kaempferol-3-O-rutinoside; 0.87%), Lucenin-2 (0.93%), 7,8-Epoxylanostan-11-ol,3-acetoxy (1.48%), Phytofluene (1.00%), Prosta-5,8(12),13-trien-1-oic acid, 15-hydroxy-9-oxo, methyl ester, (5Z,13E,15S) (0.68%), 2,6,10,14,18,22-Tetracosahexaene, 2,6,10,15,19,23-hexamethyl (2.07%), Nonacosane (0.76%), Stigmast-5-en-3-ol, 3β ( β-Sitosterol; 0.87%) and Octadecane, 3-ethyl-5-(2-ethylbutyl) (0.55%).


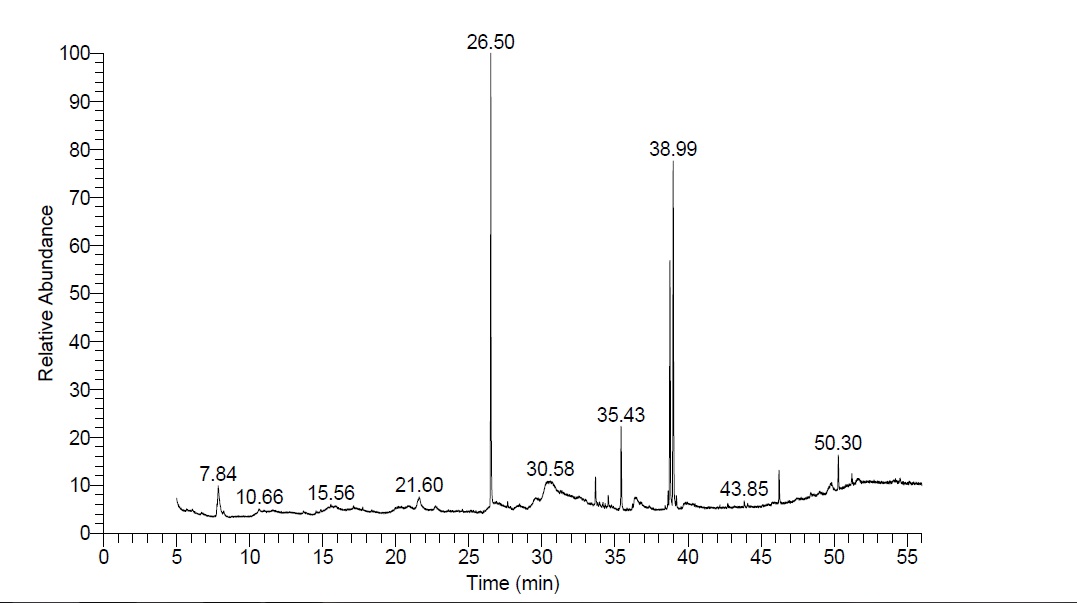


**Figure (1):** GC-MS chromatogram of *P. granatum* juice.

**Table S2:** Identification of phytochemical compounds by GC-MS in pomegranate juice.

| **Peak** | **Retention time** | **Name of the compound** | **Molecular**  **Formula** | **Molecular**  **Weight** | **Area %** |
| --- | --- | --- | --- | --- | --- |
| **1** | 6.23 min | 3,5-Dihydroxy-6-methyl-2,3-dihydro-4H-pyran-4-one | C_6_H_8_O_4_ | 144 | 5.76 |
| **2** | 7.75 min | 1,3-Cyclopentadiene,5-(1-methylethylidene) | C_8_H_10_ | 106 | 0.47 |
| **3** | 8.22 min | D-Fructose, diethyl mercaptal, pentaacetate | C_20_H_32_O_10_S_2_ | 496 | 0.35 |
| **4** | 10.06 min | Pyrogallol | C_6_H_6_O_3_ | 126 | 3.48 |
| **5** | 10.62 min | 1,3-Dioxocane, 2-pentadecyl | C_21_H_42_O_2_ | 326 | 0.55 |
| **6** | 12.56 min | α-Pinene | C_10_H_16_ | 136 | 0.68 |
| **7** | 15.55 min | 18,19-Secoyohimban-19-oic acid, 16,17,20,21-tetradehydro16-(hydroxymethyl), methyl ester, (15á,16E) | C_21_H_24_N_2_O_3_ | 352 | 0.38 |
| **8** | 19.83 min | γ -Terpinene | C_10_H_16_ | 136 | 5.43 |
| **9** | - 1. min | Methyl-9,9,10,10-D 4-octadecanoate | C_19_H_34_D_4_O_2_ | 298 | 1.82 |
| **10** | 23.27 min | Isochiapin A | C_19_H_26_O_6_ | 350 | 0.82 |
| **11** | 27.66 min | Androsta-1,4-dien-3-one,17-hydroxy-17-methyl, (17à**)** | C_20_H_28_O_2_ | 300 | 0.32 |
| **12** | 28.31 min | 2-[5-(2-Methoxy-5-nitro-phenyl)-furan-2-ylmethylene]-6,8-dimethyl-benzo[4,5]imidazo[2,1-b]thiazol-3-one | C_23_H_17_N_3_O_5_S | 447 | 0.77 |
| **13** | 29.57 min | 4,8-Dimethyl-5'-(morpholinomethyl)-4',5'-dihydro psoralen | C_18_H_21_NO_4_ | 315 | 0.86 |
| **14** | 30.28 min | Octanoic acid | C_8_H_16_O_2_ | 144 | 1.37 |
| **15** | 30.70 min | Docosane | C_22_H_46_ | 310 | 1.13 |
| **16** | 33.68 min | Cyclopentadecanol | C_15_H_30_O | 226 | 1.70 |
| **17** | 34.18 min | Z-10-Methyl-11-tetradecen-1-ol propionate | C_18_H_34_O_2_ | 282 | 0.39 |
| **18** | 35.43 min | Hexadecanoic acid, methyl ester | C_17_H_34_O_2_ | 270 | 5.36 |
| **19** | 36.38 min | Hexadecanoic acid, 2,3-dihydroxypropyl ester | C_19_H_38_O_4_ | 330 | 1.52 |
| **20** | 36.48 min | 4-Piperidine acetic acid, 1-acetyl-5-ethyl-2-[3(2-hydroxyethyl)-1H-indol-2-yl]-à-methyl, methyl ester | C_23_H_32_N_2_O_4_ | 400 | 0.84 |
| **21** | 38.64 min | 9,12-Octadecadienoic acid, methyl ester | C_19_H_34_O_2_ | 294 | 0.83 |
| **22** | 38.77 min | 9,12,15-Octadecatrienoic acid, methyl ester, (Z,Z,Z) | C_19_H_32_O_2_ | 292 | 15.64 |
| **23** | 38.99 min | Phytol | C_20_H_40_O | 296 | 22.54 |
| **24** | 39.22 min | Octadecanoic acid, methyl ester | C_19_H_38_O_2_ | 298 | 0.60 |
| **25** | 42.73 min | Flavone 4'-OH,5-OH,7-di-O-glucoside | C_27_H_30_O_15_ | 594 | 0.87 |
| **26** | 44.09 min | Lucenin 2 | C_27_H_30_O_16_ | 610 | 0.93 |
| **27** | 49.64 min | 7,8-Epoxylanostan-11-ol, 3-acetoxy | C_32_H_54_O_4_ | 502 | 1.48 |
| **28** | 49.77 min | Phytofluene | C_40_H_62_ | 542 | 1.00 |
| **29** | 49.83 min | Prosta-5,8(12),13-trien-1-oic acid, 15-hydroxy-9-oxo, methyl ester, (5Z,13E,15S) | C_21_H_32_O_4_ | 348 | 0.68 |
| **30** | 50.30 min | 2,6,10,14,18,22-Tetracosahexaene, 2,6,10,15,19,23-hexa methyl | C_30_H_50_ | 410 | 2.07 |
| **31** | 51.22 min | Nonacosane | C_29_H_60_ | 408 | 0.76 |
| **32** | 51.59 min | Stigmast-5-en-3-ol, (3á) | C_29_H_50_O | 414 | 0.87 |
| **33** | 54.53 min | Octadecane, 3-ethyl-5-(2-ethylbutyl) | C_26_H_54_ | 366 | 0.55 |

References:

1. Faria A, Monteiro R, Mateus N, Azevedo I, Calhau C: **Effect of pomegranate (Punica granatum) juice intake on hepatic oxidative stress**. *Eur J Nutr* 2007, **46**(5):271-278.
